# Alcohol consumption in early adolescence: Associations with sociodemographic and psychosocial factors according to gender

**DOI:** 10.1371/journal.pone.0245597

**Published:** 2021-01-15

**Authors:** Camille Pedroni, Maud Dujeu, Thérésa Lebacq, Véronique Desnouck, Emma Holmberg, Katia Castetbon

**Affiliations:** 1 Service d'Information, Promotion, Éducation Santé (SIPES), Research Centre in “Epidemiology, Biostatistics and Clinical Research”, School of Public Health, Université Libre de Bruxelles (ULB), Brussels, Belgium; 2 Research Centre in “Social Approaches to Health”, School of Public Health, Université Libre de Bruxelles (ULB), Brussels, Belgium; Universite de Bretagne Occidentale, FRANCE

## Abstract

**Introduction:**

Early alcohol consumption can irreversible damage the adolescents’ brain and may affect their quality of life. In order to better prevent such a deleterious behaviour, knowing its determinants is needed. So far, only few studies among adolescents aged <15 years exist, of which the majority failed to include gender differences. Therefore, the aim of this study was to investigate whether gender differences in the association between alcohol use and sociodemographic and psychosocial characteristics among 10-14-year olds exist.

**Methods:**

Data came from the 2018 *Health Behaviour in School-Aged Children* (HBSC) study conducted in French-speaking schools of Belgium. The sample analysed here comprised 4,364 10-14-year olds from the Walloon Region. Associations of the recent alcohol consumption (at least one glass during the past month) with sociodemographic and psychosocial characteristics were estimated using gender-stratified multivariable logistic regression modelling.

**Results:**

Prevalence of early alcohol consumption was 14% (boys: 16%; girls: 12%). Migration status and family affluence scale (FAS) were associated with early alcohol consumption only in boys. Second-generation immigrant boys (vs. natives: OR = 0.66 [0.47–0.92]) and boys from “low” FAS families (vs. “high”: OR = 0.56 [0.32–0.98]) or “medium” FAS (vs. “high”: OR = 0.63 [0.43–0.92]) were less likely to have consumed alcohol in the past month. In both genders, alcohol consumption was positively associated with age and inversely associated with school satisfaction and family support. No association was observed with family structure, peer support and life satisfaction in the multivariable models.

**Conclusion:**

Our findings showed that gender differences may exist in the determinants of alcohol consumption among young adolescents. They will contribute to the development of public health policies and actions for the most vulnerable adolescents, which should take gender differences into account.

## 1. Introduction

Adolescence is a key period in life characterized by many social, physical, physiological and psychological changes which may make adolescents particularly vulnerable to the adverse consequences of risk-taking behaviours, including alcohol use [1]. A report based on the 2014 *Health Behaviour in School Aged Children* (HBSC) survey data in 36 countries and regions in Europe and North-America [2] has shown that among 15-year-old adolescents, 28% had first started consuming alcohol at age 13 or younger (31% of boys and 25% of girls). Boys were more likely to report early alcohol initiation in most countries and regions. In the French-speaking schools of Belgium (Walloon Region and Brussels-Capital Region), one quarter of boys and one girl in five had consumed alcohol at age 13 or younger [2].

An “early alcohol consumption” is generally defined as having drunk before the age of 15 [3]. Starting to drink alcohol at such a very young age carries a variety of specific health risks, especially for the brain development [4, 5]. In addition, early alcohol use, when compared with an initiation in later adolescence, is associated with a raise in the frequency and in the quantity of alcohol consumed, and with more frequent alcohol-related troubles in later adolescence and early adulthood [6, 7]. However, while drinking habits are well documented in adolescents aged 15 years and older, studies on drinking habits in adolescents under the age of 15 are scarce, despite the specific risk of negative health-related consequences in this age group [8, 9]. As a result, the determinants of early alcohol use are barely known. These determinants are related to five domains: individual, family, peer, school and community [10]. The family- and peer-domains have been most studied [11–13]. For instance, parents’ and friends’ alcohol use, peer encouragement toward consumption, or low parental monitoring have been identified as risk factors for early alcohol use [11–13]. The risk factors related to the other three domains have been much less studied.

Moreover, gender disparities in various health and risk behaviours in adolescence have been identified [14–16]. Another HBSC research showed, among other disparities, that girls were more likely than boys to report high levels of perceived peer support, high satisfaction with school, ate more fruits, but described fair or poor health and lower life satisfaction [14]. In turn, boys were generally reporting more positive relationships, and physical activities, but also more frequent injuries and sedentary behaviours [14]. Regarding drinking behaviors, gender differencies have been highlighted in the determinants of early alcohol use. For instance, having a secure attachment to parents and to peers was a protective factor against early alcohol use but only for girls [17]. Similarly, among girls only, a higher level of perceived parental monitoring was associated with lower odds of early alcohol consumption [18]. However, for associations related to the other three domains (individual, school and community), too few data exist to draw robust conclusions on gender-related derterminants.

In order to complete the knowledge on this topic, the aim of this study was to identify potential gender differences in the associations between alcohol use and sociodemographic and psychosocial characteristics among adolescents aged 10 to 14 years. This knowledge will be helpful to the development of actions targeted to young consumers of alcohol, to avoid serious adverse impact on their current and future quality of life.

## 2. Materials and methods

### 2.1. Sample

We used data collected for the 2018 HBSC cross-sectional survey conducted in the French-speaking schools of Belgium. The HBSC study is conducted in almost 50 countries and regions using an international standardized protocol [19]. The study is undertaken every four years in the French-speaking schools of Belgium (Walloon Region and Brussels-Capital Region). Children and adolescents were asked about their health status, wellbeing and health-related behaviours through self-administered questionnaires they filled out in the classroom according to a standardized procedure, and treated as confidential and anonymous [20]. The protocol was approved by the educational authorities of each school network (private and public) and by the ethics committee of the Faculty of Psychology of the *Université libre de Bruxelles (ethics opinion n°032/2017)*.

A two-stage random sample was used. First, schools were randomly selected from an official list of all schools stratified per province and school network, using an allocation proportional to the school population size of each province and network. Among the 807 schools invited to participate, 266 took part in the survey, with a participation rate of 33%. Secondly, one class per grade was randomly selected in the participating schools. All selected classes were willing to participate.

In an operational point of view, a letter introducing the survey was sent to the head of each selected school. This letter contained a form to be completed to notify agreement or refusal. The schools for which no feedback was received were contacted by phone or e-mail. Then, each participating school filled out informations about the classes (number of students, education branch…) which was used to randomly select them. In addition, each school was asked to designate a coordinator to handle the distribution and retrieval of the survey materials (survey questionaires, information letters, general instructions…) and to transmit them to the research team.

Adolescents and their parents received an information letter about the study objectives, the topics covered, the confidentiality and anonymity of the data collected, and their right to refuse to participate. Parents could express their refusal for the participation of their child. On the day of the questionnaire completion, adolescents also still had the opportunity to refuse to participate in the survey. The sample included all students in the selected classes, who were present on the day of questionnaire completion and who agreed to fill it in. In all selected classes, 87.0% of the students agreed to participate and eventually completed the questionnaire.

**Fig 1:**
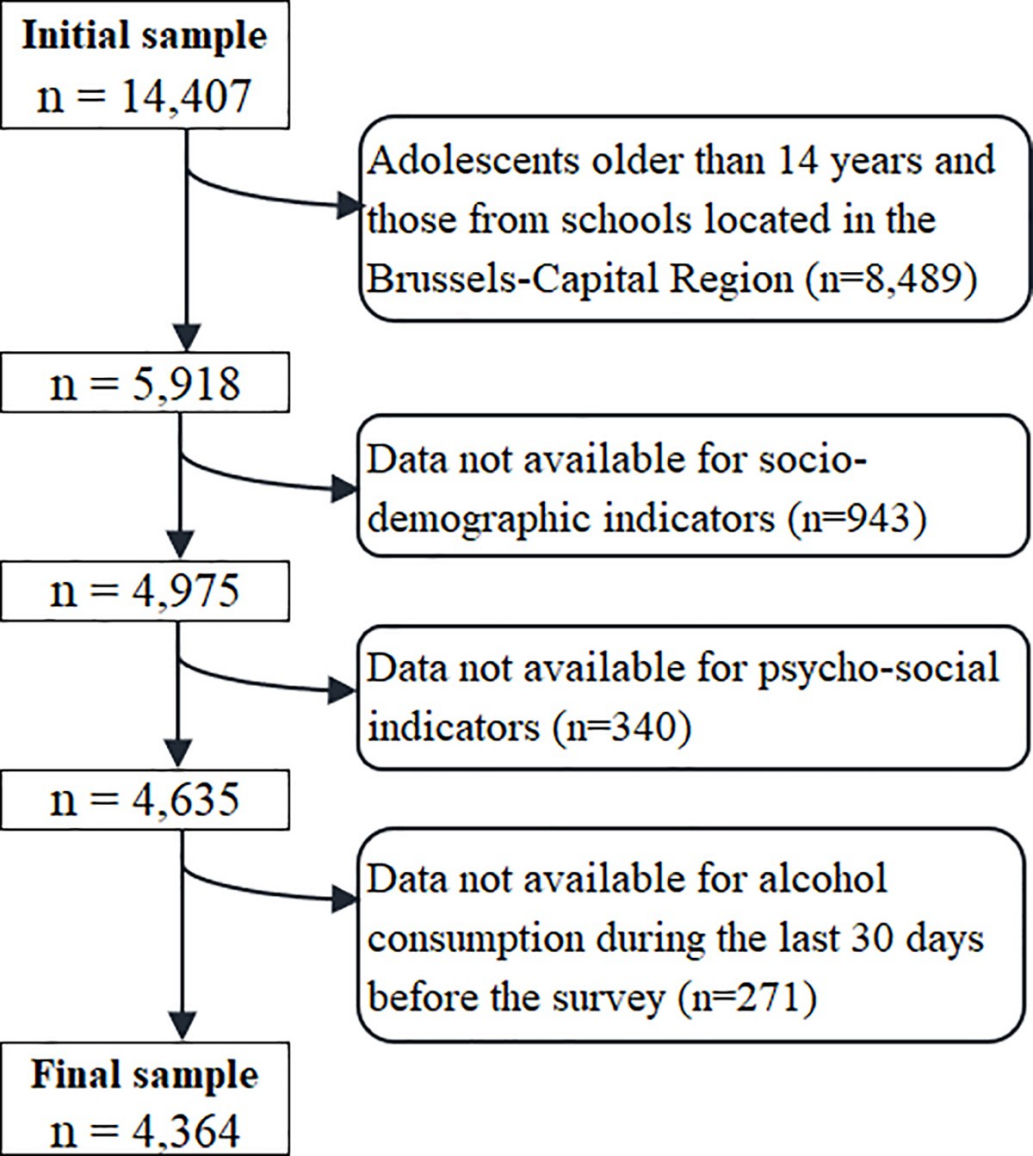
Flow chart of inclusion in analyses.

The entire 2018 HBSC sample was composed of 14,407 adolescents of 10 to 21 years of age **(Fig 1)**. Adolescents older than 14 years of age and those who attended schools in the Brussels-Capital Region were excluded from the study sample (n = 8,489). Since the two regions include very different population structure and lifestyle (in relation with socioeconomic and cultural characteristics), analyses were here restricted to Wallonia. Those who had missing values for one or more study variables were also excluded from the analyses (n = 1,554). The final sample therefore included 4,364 adolescents aged 10 to 14 years from Walloon schools **(Fig 1)**.

### 2.2. Measures

#### 2.2.1 Alcohol consumption

The question on alcohol consumption was: “On how many days (if any) have you drunk alcohol in the last 30 days (at least a glass of beer, wine, cocktail, aperitif…)?” Seven-answer options ranged from “never” to “30 days” [21]. The parenthesis was added to avoid that only sipping was accounted for by the respondants, since it has previously been shown that the youngest not really drink full glasses but only take small amount of alhoholic beverages [22]. Respondents were classified into two categories: adolescents who consumed alcohol at least one day during the last 30 days *versus* those who did not consume alcohol during the last 30 days.

#### 2.2.2 Family & peer support

Family and peer support was assessed through a four-item scale from the « *Multidimensional Scale of Perceived Social Support* » (MPSS), whose internal coherence and factorial validity have been demonstrated, especially among adolescents [23, 24]. Family support scale included statements such as “I can talk about my problems with my family” and the peer support scale, “I can count on my friends when things go wrong”. For both scales, a five-point Likert scale ranging from “strongly agree” to “strongly disagree” was given for each item. The four-item scores were summed and the sum was divided into three categories: “low” support (1 to 2.9), “moderate” support (3 to 5) and “high” support (5.1 to 7) [25].

#### 2.2.3 School satisfaction

School satisfaction was measured by the question: “How do you feel about school at present?”, response options were: “I like it a lot”, “I like it a bit”, “I do not like it very much” or “I do not like it at all” [21].

#### 2.2.4 Life satisfaction

Adolescents’ life satisfaction was assessed through the adapted version of the Cantril ladder graduated from 0 to 10, with the value 10 equivalent to "the best life possible" and the value 0 to "the worst life possible" [26]. A binary variable was created: “low” life satisfaction (from 0 to 5) *versus* “average to high” life satisfaction (from 6 to 10) [21].

#### 2.2.5 Socio-demographic variables

Socio-demographic factors we included were: gender, age (10–12 years/13-14 years) and family structure (two-parent families/blended families/one-parent families).

Adolescents were asked to indicate their country of birth and that of their parents. A “migration status” indicator was created and divided into three categories: the “first generation immigrants” (adolescents born abroad and whose parents were both not born in Belgium); the “second generation immigrants” (those born in Belgium who had at least one parent born abroad); and the “natives” (those who had both parents born in Belgium).

In addition, the adolescents’ socio-economic status was assessed through the revised Family Affluence Scale (FAS), which is composed of six items and has been previously validated [27]. Individual FAS scores ranged from 0 to 13; they were subject to a ridit transformation and classified into three groups, i.e. “low”, “medium”, and “high”, corresponding to the 20% of adolescents with the lowest FAS scores, 60% of adolescents with intermediate scores, and 20% of adolescents with the highest FAS scores, respectively.

### 2.3. Statistical analyses

To improve the estimates’ representativeness, individual weighting factors were calculated according to the inclusion probability of schools in the initial sample, schools characteristics (size, province, network and socioeconomic status) and the differences observed between the adolescents who took part in the survey and those of the reference population in terms of school grade and gender. These weighting factors as well as the sample design were taken into account in all the statistical analyses.

After the description of the sample, univariate analyses were performed to identify characteristics and behaviours associated with adolescents’ alcohol consumption during the last 30 days before the survey. Statistical associations were estimated using Pearson’s chi-square tests (Rao & Scott correction for the sampling plan). Odds ratio (OR) and 95% confidence intervals [95%CI] were calculated using logistic regressions [28]. All indicators associated with alcohol consumption in the univariate analyses with a P-value<20% were included in the multivariable models. Inter-correlations between explanatory variables were tested using Cramer’s V test; all values were lower than 0.25 (**S1 Appendix**). All statistical analyses were performed using Stata^®^ V.14.

## 3. Results

**Table 1** presents the characteristics of the sample, overall and by gender. Alcohol use during the month before the interview was higher in boys than in girls. The proportion of adolescents feeling supported by their family was higher in boys (83%) than in girls (76%). The opposite situation was observed for peer support (boys: 66%; girls: 76%). About a third liked school a lot (boys: 27%; girls: 35%). Almost all had a high level of life satisfaction (boys: 92%; girls: 87%) **(Table 1)**.

**Table 1:** Characteristics of the sample overall and by gender (n = 4,364). HBSC, French-speaking Walloon schools in Belgium, 2018.

	Overall sample		Boys		Girls		p
	(n = 4,364)		(n = 2,144)		(n = 2,220)		
	n	%	n	%	n	%	
**Alcohol use during the last 30 days before the survey**							0.002
** **No	3744	86.8	1795	83.7	1949	87.8	
** **Yes	620	14.2	349	16.3	271	12.2	
**Age**							0.25
** **10–12 years	2499	57.3	1251	58.4	1248	56.2	
** **13–14 years	1865	42.7	893	41.6	972	43.8	
**Migration status**							0.64
** **Natives	2904	66.5	1416	66.0	1488	67.0	
** **Second-generation immigrants	1121	25.7	552	25.8	569	25.6	
** **First-generation immigrants	339	7.8	176	8.2	163	7.3	
**Family affluence scale (FAS)**							0.63
** **High	1033	23.7	505	23.6	528	23.8	
** **Medium	2680	61.4	1332	62.1	1348	60.7	
** **Low	651	14.9	307	14.1	344	15.5	
**Family structure**							0.12
** **Two-parent families	2922	67.0	1406	65.6	1516	68.3	
** **Blended families	621	14.2	323	15.1	298	13.4	
** **One-parent families	821	18.8	415	19.3	406	18.3	
**Family support**							<0.001
** **High	3460	79.3	1778	82.9	1682	75.8	
** **Moderate	640	14.6	262	12.2	378	17.0	
** **Low	264	6.0	104	4.9	160	7.2	
**Peer support**							<0.001
** **High	3109	71.2	1425	66.4	1684	75.9	
** **Moderate	837	19.2	475	22.2	362	16.3	
** **Low	418	9.6	244	11.4	174	7.8	
**School satisfaction**							<0.001
** **Like school a lot	1363	31.2	585	27.3	778	35.0	
** **Like school a bit	1928	44.2	956	44.6	972	43.8	
** **Does not like school very much	720	16.5	380	17.7	340	15.3	
** **Does not like school at all	353	8.1	223	10.4	130	5.8	
**Life satisfaction**							<0.001
** **Moderate to high	3907	89.6	1981	92.4	1925	86.7	
** **Low	457	10.4	163	7.6	294	13.3	

All indicators are described in the methods section.

Estimations are weighted; all models take the sample design into account.

In univariate analyses, in boys, all characteristics and behaviours except family structure and peer support, were significantly associated with alcohol consumption **(Table 2)**. In girls, only age, family support, school satisfaction and life satisfaction were significantly associated with alcohol consumption. In the multivariable logistic regression models **(Table 2)**, age, family support and school satisfaction were significantly associated with alcohol consumption in both boys and girls. Adolescents aged 13–14 years (vs. 10–12 years: boys: ORa = 3.14 [2.34–4.22]; girls: ORa = 4.45 [3.15–6.28]) were more likely to have consumed alcohol in the last 30 days preceding the survey. Associations between family support and early alcohol consumption were a little different between boys and girls. Girls who felt “moderate” (ORa = 1.59 [1.04–2.43]) or “low” (ORa = 1.94 [1.17–3.21]) support by their family were more likely to have consumed alcohol during the past month compared with the “high” category. In boys, this association was only statistically significant in the “moderate” *versus* the “high” categories (ORa = 1.58 [1.13–2.22]). In both boys and girls, school satisfaction was inversely associated with alcohol consumption. Compared with those who liked school very much, those who liked school a bit (boys: ORa = 2.15 [1.45–3.20]; girls: ORa = 1.68 [1.08–2.60]), those who did not like school very much (boys: ORaa = 2.33 [1.25–4.33]; girls: ORa = 2.12 [1.30–3.46]), and those who did not like school at all (boys: ORa = 4.88 [2.85–8.36]; girls: ORa = 3.35 [1.85–6.08]), were more likely to have consumed alcohol in the last 30 days before the survey **(Table 2)**.

**Table 2:** Characteristics and behaviors associated with alcohol consumption during the last 30 days before the survey among adolescents aged 10 to 14. HBSC, French-speaking Walloon schools in Belgium, 2018.

	Boys (n = 2,144)						Girls (n = 2,220)					
	n	%	Crude OR (95%CI)	p	Adujsted OR (95%CI)	p	n	%	Crude OR (95%CI)	p	Adjusted OR (95%CI)	p
**Age**				<0.001		<0.001				<0.001		<0.001
** **10–12 years	1251	9.1	1		1		1248	4.8	1		1	
** **13–14 years	893	26.4	3.56 (2.68–4.73)		3.14 (2.34–4.22)		972	21.6	5.46 (3.93–7.57)		4.45 (3.15–6.28)	
**Migration status**				0.02		0.049				0.60		0.57
** **Natives	1416	18.0	1		1		1488	12.8	1		1	
** **Second-generation immigrants	552	12.9	0.68 (0.51–0.90)		0.66 (0.47–0.92)		569	10.9	0.84 (0.60–1.19)		0.80 (0.54–1.20)	
** **First-generation immigrants	176	13.0	0.68 (0.42–1.09)		0.74 (0.45–1.20)		163	11.5	0.89 (0.48–1.65)		0.83 (0.44–1.56)	
**Family affluence scale (FAS)**				0.03		0.04				0.62		0.22
** **High	505	21.0	1		1		528	13.0	1		1	
** **Medium	1332	15.3	0.68 (0.49–0.93)		0.63 (0.43–0.92)		1348	12.3	0.94 (0.65–1.37)		1.02 (0.68–1.55)	
** **Low	307	13.0	0.56 (0.35–0.90)		0.56 (0.32–0.98)		344	10.5	0.80 (0.48–1.30)		0.71 (0.40–1.23)	
**Family structure**				0.24		0.38				0.41		0.97
** **Two-parent families	1406	15.8	1		1		1516	11.7	1		1	
** **Blended families	323	20.1	1.35 (0.93–1.95)		1.26 (0.86–1.87)		298	14.6	1.28 (0.88–1.87)		1.05 (0.64–1.71)	
** **One-parent families	415	15.2	0.95 (0.70–1.29)		0.94 (0.67–1.31)		406	12.2	1.04 (0.73–1.50)		1.00 (0.66–1.52)	
**Family support**				<0.001		0.01				<0.001		0.01
** **High	1778	14.7	1		1		1682	9.7	1		1	
** **Moderate	262	25.3	1.96 (1.44–2.66)		1.58 (1.13–2.22)		378	19.4	2.25 (1.53–3.29)		1.59 (1.04–2.43)	
** **Low	104	20.5	1.49 (0.89–2.50)		1.41 (0.91–2.20)		160	21.6	2.57 (1.62–4.08)		1.94 (1.17–3.21)	
**Peer support**				0.55		0.47				0.70		0.41
** **High	1425	15.7	1		1		1684	12.0.	1		1	
** **Moderate	475	17.1	1.11 (0.81–1.52)		0.82 (0.59–1.13)		362	13.6	1.16 (0.81–1.66)		0.90 (0.58–1.40)	
** **Low	244	18.5	1.22 (0.82–1.82)		0.97 (0.65–1.45)		174	11.3	0.94 (0.49–1.80)		0.64 (0.33–1.24)	
**School satisfaction**				<0.001		<0.001				<0.001		<0.001
** **Like school a lot	585	7.4	1		1		778	6.1	1		1	
** **Like school a bit	956	16.5	2.49 (1.71–3.62)		2.15 (1.45–3.20)		972	12.6	2.23 (1.51–3.29)		1.68 (1.08–2.60)	
** **Does not like school very much	380	20.0	3.15 (1.76–5.66)		2.33 (1.25–4.33)		340	18.1	3.42 (2.09–5.59)		2.12 (1.30–3.46)	
** **Does not like school at all	223	32.8	6.14 (3.61–10.45)		4.88 (2.85–8.36)		130	29.8	6.55 (3.80–11.27)		3.35 (1.85–6.08)	
**Life satisfaction**				0.02		0.29				<0.001		0.10
** **Moderate to High	1981	15.7	1		1		1925	10.7	1		1	
** **Low	163	23.3	1.62 (1.07–2.46)		1.29 (0.80–2.07)		294	21.8	2.31 (1.70–3.16)		1.37 (0.94–2.00)	

All indicators are described in the methods section.

Estimations are weighted; all models take the sample design into account.

In boys only, migration status and FAS were significantly associated with early alcohol consumption **(Table 2)**. Boys from “low” FAS (ORa = 0.56 [0.32–0.98]) or “medium” FAS families (ORa = 0.63 [0.43–0.92]) compared with those from “high” FAS families were less likely to have consumed alcohol during the past month. Lastly, second-generation immigrant boys (ORa = 0.66 [0.47–0.92]) were less likely to have consumed alcohol compared to natives **(Table 2)**.

## 4. Discussion

The aim of this study was to identify potential gender differences in the correlates of alcohol consumption in early adolescence. We did not find any gender difference with the psychosocial indicators. The main gender differences were found for migration status and FAS, which were significantly associated with alcohol consumption in boys only. Indeed, our results showed that second-generation immigrant boys were less likely to have consumed alcohol compared to natives. Consistently with these findings in boys, a Dutch longitudinal study in adolescents age 10–14, found that being a native was a predictor of early alcohol use [9]. Some cross-sectional studies came to the same conclusion [29]. However, the results could differ depending on the country of origin of the immigrant adolescent. In fact, alcohol consumption in adolescents is closely linked to religion as well as to the culture and customs of the country of origin. For instance, prevalence of alcohol consumption is generally higher in adolescents from Eastern European countries compared with those from Northern Europe [30, 31].

Concerning FAS, boys from “low” FAS or “medium” FAS families compared with those from “high” FAS families were less likely to have consumed alcohol during the past month. In a recent review, no clear pattern of associations between socio-economic status (SES) and alcohol consumption was identified [32]. A meta-analysis focusing on adolescents aged 10–15, indicated that the lower the SES, the higher the prevalence of alcohol use. However, results were inconclusive when regarding European studies only [33]. Some authors have suggested that these conflicting results between studies may be partly due to the measures used to assess the SES (e.g. parents’ education level, occupational status, family affluence or income) and the drinking behavior assessed (sipping, lifetime use, binge drinking, etc.) [22, 34–36]. For example, several studies have shown that alcohol misuse was more prevalent among adolescents with low SES, whereas experimental alcohol use occurred more often among those with high SES [34, 35]. No study focusing on gender differences in the association between SES and early alcohol consumption was found.

The influence of the family structure on adolescents’ substance use has been widely studied [37, 38]. It has been argued that living in two-parent families could play a protective role on adolescent substance use. This hypothesis was confirmed by many cross-sectional and longitudinal studies [29, 39]. Very few studies have focused on potential gender differences however. One study, in 11-15-year-old Flemish adolescents, showed that boys from blended families and girls from one-parent families were more likely to consume alcohol at least once a week in comparison with adolescents from two-parent families [40]. In our study, even in the univariate analysis, no association was found at all. Further research therefore is needed to confirm whether any relationship between early alcohol use and family structure exists.

The influence of parenting factors (parents drinking behaviour, parenting style, parent’s rules toward alcohol, etc.) on adolescent alcohol use has also been widely investigated in previous research [40–42]. Furthermore, a recent systematic review and meta-analysis of longitudinal studies focusing on modifiable parenting factors in adolescents aged 12–18 concluded that parental support was a protective factor related to both alcohol initiation and levels of later use [13]. Other studies among younger adolescents came to the same conclusion [42]. We also found an association between parental support and alcohol consumption in our study, with little difference between boys and girls.

In our study, in both genders, we did not find an association between peer support and early alcohol use. In the literature, several studies have examined the role on early drinking of certain determinants related to peer influence and some, such as peer approval, have been identified as predictors for starting to drink [11]. However, among the few studies that have examined this association, findings are divergent. For example, the Welsh study [43] found that peer support was a protective factor against binge drinking among 11-15-year-old adolescents, whereas another study among 15 year olds in the U.S. [12], showed that peer support neither contributed to nor protected against the alcohol use (based on alcohol consumption frequency during the last 30 days). These divergent findings might be due to a lack of comparability in the tools used to assess peer support or due to interactions with other indicators not included in this study such as peer involvement in the alcohol consumption. Further research, using a qualitative assessment, is highly needed to elucidate the role of peer support on early alcohol use.

We found a negative association in both genders between school satisfaction and alcohol consumption during the past month prior the survey. Two cross-sectional studies showed that 11-15-year olds who liked school were less likely to drink alcohol [43, 44]. In contrast, two other cross-sectional studies found no association [29, 41]. However, the adolescents included in these two latter were older (15 to 20 years of age) than those included in ours. It therefore may be assumed that school satisfaction may have an influence on alcohol consumption only among young adolescents.

Several studies have found a negative association between life satisfaction and early alcohol use [10, 43]. For example, an American study showed that 10-17-year olds with “low” life satisfaction were also more likely to consume alcohol in conjunction with tobacco and/or marijuana [10]. In our study, in both gender, we found a significant association between life satisfaction and early alcohol use in univariate analysis, but this association disappeared in multivariate models, when school satisfaction was added into the models.

One strength of our study lies in the focus on adolescents from the age of 10. Although a number of studies among young adolescents identified some predictors of alcohol consumption [3, 6, 11, 17], they remain too few and inconsistent between each other to be able to draw clear conclusions about such determinants. Our study also extends previous research by investigating gender differences in the association between early alcohol use and sociodemographic and psychosocial factors. Furthermore we also examined the role in this association, of some indicators which were less studied before (peer support for example). Our findings therefore provide new information on the sociodemographic and psychosocial factors possibly involved in alcohol use in early adolescence in both genders. Finally, we used a large randomized sample, which is another strength to our study.

At the same time, some limitations should also be considered. Firstly, in our cross-sectional study, no causal link can be inferred from the observations made. Indeed, bilateral associations could exist between early alcohol consumption and some characteristics such as family support. We can hypothesize that adolescents with “low” family support are more likely to drink alcohol, but alcohol consumption may also make adolescents less close to their family and therefore, feeling less supported by them. Secondly, all data collected were self-reported which can be a source of declaration bias [45]. One of them is the social desirability bias which may lead, among others, to an underestimation of alcohol consumption [45, 46]. Another possible bias is linked to memorization due to the fact that we have asked adolescents to report their alcohol consumption during the past month. Nevertheless, some evidence is available that short recall periods like we used here lead to more accurate reporting [45].

## 5. Conclusion

We investigated the determinants of alcohol consumption among adolescents under 15 years of age taking gender into account. This topic has been insufficiently treated so far, with the exception of family support and peer influence. Our findings therefore provide crucial new knowledge about this public health issue, since alcohol consumption in young adolescents can have serious consequences for their current and future health and quality of life.

Whereas both genders shared common risk factors (age, family support and school satisfaction), our findings have highlighted gender differences in the following sociodemographic risk factors associated with early alcohol consumption: migration status and FAS. Nevertheless, since a number of the potential determinants we have analysed here have received little attention in the previous studies, we need research on gender-specific determinants of early alcohol consumption to draw firm conclusions. This knowledge will help the developers of public health policies and actions to better target and adapt messages to the most vulnerable adolescents.

## Supporting information

S1 Appendix Inter-correlations between variables included in the study. (DOCX)
