# Supplementary material for: Alcohol consumption in early adolescence: Associations with sociodemographic and psychosocial factors according to gender
Source: PLoS One. 2021 Jan 15;16(1):e0245597. doi: 10.1371/journal.pone.0245597 (PMC7810307; doi:10.1371/journal.pone.0245597)
Supplement: S1 Appendix — (DOCX) [file pone.0245597.s001.docx]

**S1 Appendix.** Inter-correlations between variables included in the study.

| Variables |  | Cramer’s V |
| --- | --- | --- |
| Gender | Age  Migration status  FAS  Family structure  Family support  Peer support  School satisfaction  Life satisfaction | 0.02  0.01  0.02  0.01  0.04  0.08  0.09  0.09 |
| Age | Migration status  FAS  Family structure  Family support  Peer support  School satisfaction  Life satisfaction | 0.00  0.02  0.06  0.11  0.03  0.19  0.06 |
| Migration status | FAS  Family structure  Family support  Peer support  School satisfaction  Life satisfaction | 0.09  0.07  0.02  0.03  0.03  0.04 |
| Family affluence scale (FAS) | Family structure  Family support  Peer support  School satisfaction  Life satisfaction | 0.14  0.07  0.05  0.03  0.13 |
| Family structure | Family support  Peer support  School satisfaction  Life satisfaction | 0.09  0.03  0.05  0.12 |
| Family support | Peer support  School satisfaction  Life satisfaction | 0.19  0.12  0.23 |
| Peer support | School satisfaction  Life satisfaction | 0.09  0.16 |
| School satisfaction | Life satisfaction | 0.18 |
